# Pulsed TM-YAG laser (Thulio®): a new weapon in endourologists’ hand in the conservative management of imperative cases of Upper Tract Urothelial Carcinoma (UTUC)

**DOI:** 10.1590/S1677-5538.IBJU.2024.0653

**Published:** 2025-01-22

**Authors:** Riccardo Scalia, Stefano Gisone, Rebeca Escobar, Silvia Proietti, Franco Gaboardi, Guido Giusti

**Affiliations:** 1 ETCE San Raffaele Hospital Department of Urology Milan Italy Department of Urology, San Raffaele Hospital, ETCE (European Training Center in Endourology), Milan, Italy

## Abstract

**Introduction:**

Urothelial carcinomas (UC) represent the sixth most common tumor by incidence, involving the lower or upper urinary tracts (UTUC) (1). High-risk patients should be treated by nephroureterectomy with complete bladder cuff excision (2), conservative approach is reserved for low-risk UTUCs and/or imperative cases (3).

**Materials and Methods:**

We present a 70-year-old male patient, smoker, with history of urothelial carcinoma. He underwent distal ureterectomy with ileal replacement in April 2019. Since then, he has developed several UTUC recurrences bilaterally and in the bladder, which have been treated conservatively. In August 2023, CT- scan showed multiple recurrences in the left kidney and ureter. Hence, on November 2023, we performed cystoscopy, monopolar resection of bladder tumor and bilateral flexible ureteroscopy (fURS) with pulsed thulium:YAG (p-Tm:YAG) ablation of the tumors.

We performed a no-touch technique fURS with Video Uretero-Renoscope FLEX-XC1 by Storz. After this, we placed an ureteral access sheath and then a biopsy by using a tipless basket.

The laser fiber used was 272 μm and the laser settings were 0.8 J – 10 Hz – Long pulse Ablation (10 W).

**Results:**

The pathological results showed UTUC bilaterally and high-grade UC in the bladder. Then, he underwent intravenous therapy with enfortumab - vedotin and the follow-ups, in February 2024 and June 2024, showed no evidence of recurrences at the multiple biopsies.

**Conclusion:**

The p-Tm:YAG laser can be considered a valid alternative option for the conservative treatment of UTUCs. With that said, stringent follow-up remains a mainstay in the conservative treatment of imperative cases of UTUC.

Available at: http://www.intbrazjurol.com.br/video-section/20240653_Scalia_et_al
